# Association between cardiopulmonary bypass time and mortality among patients with acute respiratory distress syndrome after cardiac surgery

**DOI:** 10.1186/s12872-023-03664-3

**Published:** 2023-12-19

**Authors:** Jiaxin Hu, Yan liu, Lixue Huang, Man Song, Guangfa Zhu

**Affiliations:** 1 Department of Respiratory and Critical Care Medicine, Beijing Anzhen Hospital, Capital Medical University, No.2 Anzhen Road, Beijing, 100029 PR China; 2 Department of Infectious Diseases, Beijing Anzhen Hospital, Capital Medical University, Beijing, PR China; 3 Department of Respiratory and Critical Care Medicine, Beijing Hospital, National Center of Gerontology, Institute of Geriatric Medicine, Chinese Academy of Medical Sciences, Beijing, China

**Keywords:** Cardiopulmonary bypass, Cardiac Surgery, Acute respiratory distress syndrome, Mortality prediction, Outcome

## Abstract

**Background:**

Cardiopulmonary bypass (CPB) can lead to lung injury and even acute respiratory distress syndrome (ARDS) through triggering systemic inflammatory response. The objective of this study was to investigate the impact of CPB time on clinical outcomes in patients with ARDS after cardiac surgery.

**Methods:**

Totally, patients with ARDS after cardiac surgery in Beijing Anzhen Hospital from January 2005 to December 2015 were retrospectively included and were further divided into three groups according to the median time of CPB. The primary endpoints were the ICU mortality and in-hospital mortality, and ICU and hospital stay. Restricted cubic spline (RCS), logistic regression, cox regression model, and receiver operating characteristic (ROC) curve were adopted to explore the relationship between CPB time and clinical endpoints.

**Results:**

A total of 54,217 patients underwent cardiac surgery during the above period, of whom 210 patients developed ARDS after surgery and were finally included. The ICU mortality and in-hospital mortality were 21.0% and 41.9% in all ARDS patients after cardiac surgery respectively. Patients with long CPB time (CPB time ≥ 173 min) had longer length of ICU stay (*P* = 0.011), higher ICU (*P* < 0.001) mortality and in-hospital(*P* = 0.002) mortality compared with non-CPB patients (CPB = 0). For each ten minutes increment in CPB time, the hazards of a worse outcome increased by 13.3% for ICU mortality and 9.3% for in-hospital mortality after adjusting for potential factors. ROC curves showed CPB time presented more satisfactory power to predict mortality compared with APCHEII score. The optimal cut-off value of CPB time were 160.5 min for ICU mortality and in-hospital mortality.

**Conclusions:**

Our findings demonstrated the significant prognostic value of CPB time in patients with ARDS after cardiac surgery. Longer time of CPB was associated with poorer clinical outcomes, and could be served as an indicator to predict short-term mortality in patients with ARDS after cardiac surgery.

## Introduction

Acute respiratory distress syndrome (ARDS) is an uncommon but devastating complication after cardiac surgery with the incidence is estimated to be approximately 1.14–1.15% [1]. Although significant progress has been achieved in treating ARDS including mechanical ventilation and symptomatic treatment, the mortality of ARDS after cardiac surgery still approaches 50%, and no specific biomarkers are available for those critically ill patients [2]. Recently, more and more researchers have recognized the importance of preventing the occurrence of ARDS, which is particularly necessary for patients undergoing cardiac surgery [3]. Early identification of those at higher risk is of great significance for clinicians to adopt specific prevention strategies. The risk factors associated with developing ARDS after cardiac surgery are various, such as the use of cardiopulmonary bypass (CPB), massive volume shifts, ischemia-reperfusion injury, and direct surgical strike [4]. Moreover, many cardiac patients exist chronic pulmonary or heart disease before surgery (such as, long-term smoking, chronic obstructive pulmonary disease, or poor cardiac function) [5], all of which make them especially susceptible to occur ARDS after surgery. The impact of ARDS for those patients is substantial, which is related to not only prolonged hospital length of stay, but also poorly short-term survival and increased morbidity rate of long-term physical and psychological troubles [6].

Nowadays, CPB has been widely used in cardiac surgery to temporarily replace heart and/or lung function, but it makes the lung particularly vulnerable which may be manifested as transient hypoxemia, lung injury or even developing ARDS due to exposure blood to the large synthetic, non-endothelial cell surface [7]. The reasons the lung is so susceptible to injury can be attributed partly to the alteration of pulmonary mechanics and gas exchange (such as sharply decreased bronchial arterial blood flow and accompanying ischemia-reperfusion injury) [2], and partly to the systemic inflammatory responses, including infiltration of leukocytes, increased capillary permeability, and extravasation of protein-rich fluid into the interstitium and alveolar space [8]. Our previous studies have also verified that CPB time is an independent risk factor for ARDS after cardiac surgery [9, 10].

Despite it is generally recognized that CPB is a potential risk factor for postoperative ARDS, there are few clinical studies that inspected the quantitative impact between CPB time and the clinical outcomes of those ARDS patients after cardiac surgery. Therefore, the objective of our study was to explore and quantify the association between CPB time and clinical manifestation and outcomes of patients with ARDS after cardiac surgery.

## Methods

### Study design and settings

This is an observational, retrospective, single-center study conducted by the Department of Respiratory and Critical Care Medicine and Cardiac Surgery in Beijing Anzhen Hospital, Capital Medical University, from January 2005 to December 2015. Our study had no intervention on clinical treatment of participating patients. Ethical approval was acquired from the Institutional Review Board of Beijing Anzhen Hospital (No.20,141,103), and the study protocol was registered on ClinicalTrial.gov on 03/05/2016 (NCT02759770). Each patient has signed an informed consent before enrolling into the study.

### Study population

All patients who met the criteria were screened. Inclusion criteria as follows: (1) Age > 18 years; (2) Programmed cardiac surgery with or without CPB from January 2005 to December 2015 Beijing Anzhen Hospital; (3) ARDS was diagnosed according to the criteria in Berlin definition (2012) [11]. The diagnosis was made by two clinicians independently, the patient was considered of occurring ARDS only after the confirmation by both clinicians.

All eligible patients were divided into three groups according to the median time of CPB: non-CPB group (CPB time = 0), short CPB time group (0 < CPB < 173 min), and long CPB time group (CPB 173 ≥ minutes).

### Data collection and outcomes

Demographic characteristics, comorbidities, medical treatment (include drug use, mechanical ventilation, and life support therapy), operation information (such as operation type, operation time, CPB time, and transfusion), and clinical outcomes were collected from patient’s electronic medical record. Laboratory data and Acute Physiology and Chronic Health Evaluation (APACHE) II score were collected within the first 24 h after ICU admission.

Patients were followed to discharge or death. The primary endpoint was the short-term mortality (ICU- and hospital mortality), and ICU and hospital stay. Secondary endpoints included ARDS severity, and duration of mechanical ventilation.

### Statistical analysis

Continuous variables were analyzed by one-way analysis of variance or Kruskal-Wallis test when appropriate, and the results were exhibited as mean ± standard deviation (SD) or median ± interquartile range (IQR); categorical variables were tested by chi-squared or Fisher’s test, and the results were presented as frequencies and percentages.

Restricted cubic spline model (RCS) was utilized to determine the relationship between CPB time and ICU- and hospital mortality. Univariable logistic regression analysis was conducted to evaluate risk factors of ICU mortality and in-hospital mortality, odds ratio (OR), 95% confidence interval (CI) and *P* values were demonstrated. The Kaplan–Meier (K-M) survival curves examined by log-rank test was performed to visualize the relationship between survive time and three groups. It is notable that K-M only exhibited 30-day mortality, some patients who died after 30 days were excluded from the K-M curves. In fact, most of the patients died within 30 days.

Furthermore, the Cox regression model was applied to estimate the association between CPB time and short-term mortality with the adjustments of models 1 and 2, the results were presented by the hazards ration (HR) with 95% CI. Finally, we carried out the receiver operating characteristic (ROC) curve to evaluate the ability of CPB time and APACHE II score in predicting ICU- and hospital mortality, the cut-off value was determined according to Youden index.

All above statistical analyses were conducted by SPSS 25.0 and R software 4.1.1. A two-sided probability value of < 0.05 was considered statistically significant for all results.

## Result

### Patient characteristics

**Fig. 1:**
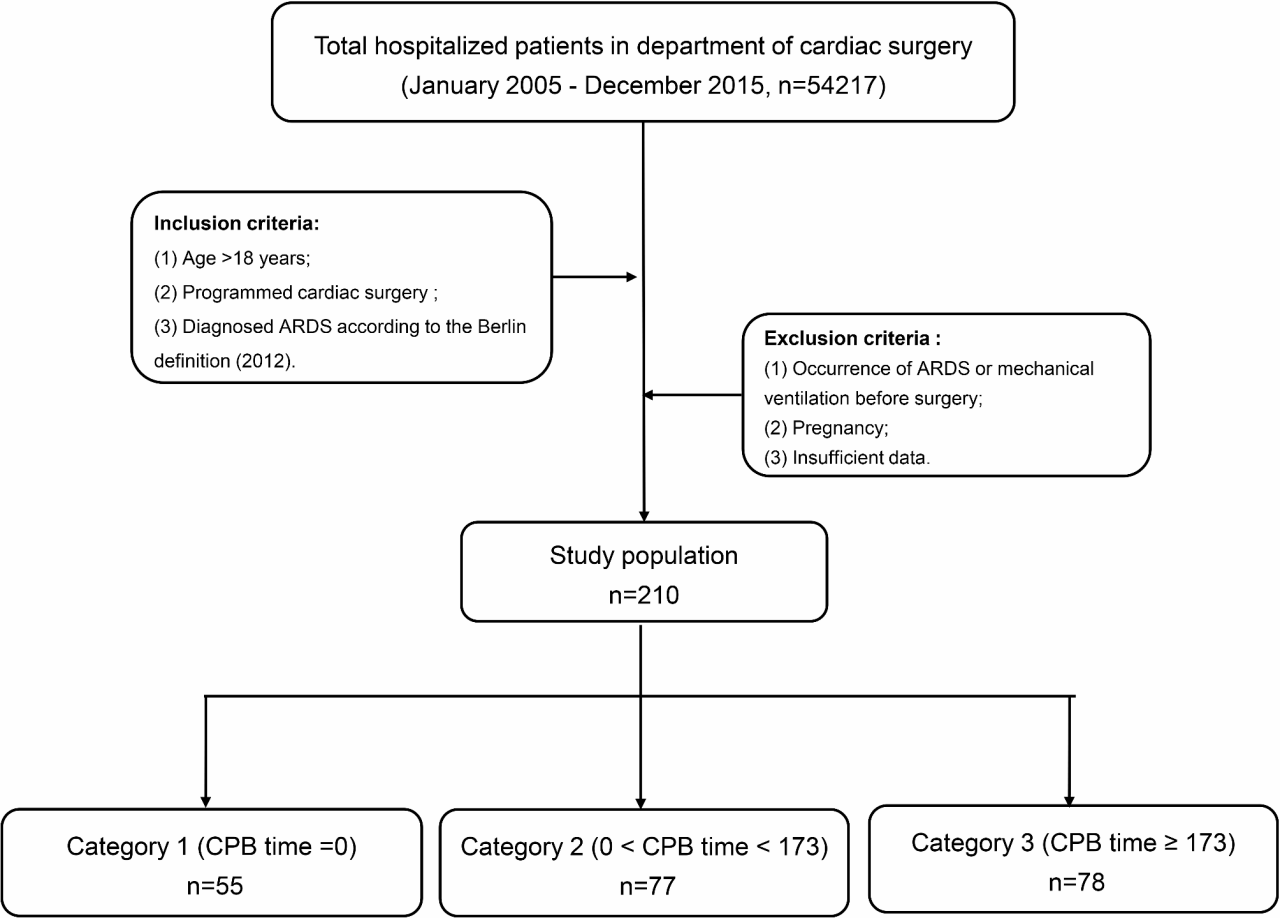
Flowchart of the study

A total of 54,217 patients undergone cardiac surgery from January 2005 to December 2015, among which, 210 patients were finally enrolled on the grounds of selection criteria (Fig. 1**).** According to the median time of CPB, 55 (26.2%), 77 (36.7%), and 78 (37.1%) patients were assigned to the group named non-CPB, short CPB time, and long CPB time respectively.

Univariate analysis of perioperative characteristics by CPB time group were separately shown in Tables 1 and 2. In total, the ICU mortality and in-hospital mortality were 21.0% and 41.9% in all ARDS patients respectively. Patients were aged 58.5 ± 13.6 years and 140 (66.7%) were males. In preoperative data, we found that patients with long CPB time.

**Table 1:** Preoperative characteristics of 210 patients with ARDS after cardiac surgery grouped by CPB time

	Total	Non-CPB	Short CPB time	Long CPB time	*P* value
Demographic data					
n (%)	210	55 (26.2)	77 (36.7)	78 (37.1)	
Age (years), mean ± SD	58.5 ± 13.6	65.7 ± 10.7	57.9 ± 13.3^a^	54.1 ± 13.8^b^	< 0.001
Male, n (%)	140 (66.7)	35 (63.6)	46 (59.7)	59 (75.6)	0.135
BMI (kg/m^2^), mean ± SD	24.7 ± 4.1	25.3 ± 3.5	23.7 ± 4.3	25.3 ± 4.1^c^	0.016
Comorbidities, n (%)					
Smoking, n (%)	80 (38.1)	22 (40.0)	23 (29.9)	35 (44.9)	0.149
Hypertension	114 (54.3)	31 (56.4)	31 (40.3)	52 (66.7)^c^	0.004
Diabetes mellitus	44 (21)	22 (40.0)	10 (13.0)^a^	12 (15.4)^b^	< 0.001
Cerebrovascular disease	27 (12.9)	8 (14.5)	11 (14.3)	8 (10.3)	0.687
Chronic lung disease	14 (6.7)	5 (9.1)	3 (3.9)	6 (7.7)	0.449
Coronary heart disease	87 (41.1)	38 (69.1)	21 (27.3) ^a^	28 (35.9) ^b^	< 0.001
Valve disease	27 (12.9)	0 (0)	17 (22.1)	10 (12.8)	< 0.001
Preoperative LVEF (%), mean ± SD	60.5 ± 8.9	60.2 ± 8.7	58.8 ± 10.1	62.5 ± 7.4^c^	0.031
NYHA grade III-IV, n (%)	75 (35.7)	23 (41.8)	30 (39.0)	22 (28.2)	0.206
Preoperative drug therapy, n (%)					
ACEI	62 (29.5)	25 (45.5)	16 (20.8)^a^	21 (26.9)	0.007
Beta-blocker	111 (52.9)	41(74.5)	35 (45.5)^a^	35 (44.9)^b^	0.001
CCB	64 (30.5)	17 (30.9)	21 (27.3)	26 (33.3)	0.712
Aspirin	24 (11.4)	15 (27.3)	5 (6.5)^a^	4 (5.1)^b^	< 0.001
Statins	68 (32.4)	31 (56.4)	19 (24.7)	18 (23.1)	< 0.001
Surgery type, n (%)					< 0.001
CABG	65 (31.1)	52 (94.5)	7 (9.1)^a^	6 (7.8)^b^	< 0.001
Valve replacement	65 (31.1)	0 (0)	42 (54.5) ^a^	23 (29.9) ^b,c^	< 0.001
Ascending aorta replacement	69 (33.0)	2 (3.6)	22 (28.6) ^a^	45 (58.4) ^b,c^	< 0.001
Other	10 (4.8)	1 (1.8)	6 (7.8)	3 (3.9)	0.252
Emergency surgery	51	9	16	26	< 0.001
Operation time (h), mean ± SD	6.2 ± 2.7	4.5 ± 1.5	5.4 ± 1.8^a^	8.1 ± 3.0^b,c^	< 0.001
Aortic clamping time (min) median (IQR)	79.0 (0-116.5)	0	79.0 (62.5–100.0)^a^	126.5 (101.2-159.3)^b,c^	< 0.001
Blood loss, (mL)	800 (600–1500)	600 (400–900)	800 (600–1000)	1200 (800–1800)^b,c^	< 0.001
RBC infusion (U)	12 (6–20)	6.0 (4.0–12.0)	12.0 (6.0-18.5)^a^	16.0(8.0–24.0) ^b^	< 0.001
FFP infusion (mL)	800 (200–1600)	400 (200–1000)	800 (300–1600)^a^	1000 (400–2050)^b^	0.001
PLT infusion (U)	0 (0–4)	0 (0–2)	0 (0–4)^a^	2 (0–4)^b^	< 0.001

^a^ Non CPB vs. short CPB time, *P* < 0.05; ^b^ short CPB time vs. long CPB time (CPB 173 ≥ minutes), *P* < 0.05; ^c^ non CPB vs. long CPB time, *P* < 0.05; Short CPB time = 0 < CPB < 173 min, long CPB time = CPB 173 ≥ minutes

LVEF = left ventricular ejection fraction, NYHA = New York Heart Association, ACEI = angiotensin converting enzyme inhibitor, CCB = calcium channel blocker, CABG = coronary artery bypass grafting, RBC = red blood cell, FFP = fresh frozen plasm

**Table 2:** Postoperative characteristics of 210 patients with ARDS after cardiac surgery, grouped by CPB time

	Total	Non CPB	Short CPB time	Long CPB time	*P* value
Laboratory data, mean ± SD					
WBC (10^9^/L)	14.8 ± 6.3	15.1 ± 4.6	14.6 ± 7.0	14.8 ± 6.8	0.931
Hb (g/L)	87.7 ± 15.4	93.7 ± 15.4	87.3 ± 15.8^a^	83.9 ± 13.8^b^	0.001
Sodium (mmol/L)	143.8 ± 7.2	139.4 ± 6.0	144.5 ± 16.3	146.2 ± 6.9^b^	0.004
Potassium (mmol/L)	4.3 ± 1.0	3.9 ± 0.8	4.2 ± 1.0	4.8 ± 0.9 ^b,c^	< 0.001
BUN (mmol/L)	32.4 ± 15.2	20.7 ± 7.9	33.2 ± 15.3^a^	40.0 ± 13.9 ^b,c^	< 0.001
Lactate (mmol/L)	8.8 ± 4.6	6.2 ± 4.2	9.3 ± 3.8^a^	10.1 ± 4.9^b^	< 0.001
Central venous pressure (cmH_2_O), mean ± SD	11.7 ± 3.4	10.7 ± 2.4	11.9 ± 3.6	12.2 ± 3.6	0.041
Postoperative LVEF (%), mean ± SD	51.3 ± 10.6	52.3 ± 9.7	51.1 ± 10.0	50.8 ± 11.7	0.697
PaO2/FIO2 ratio	131.2 ± 49.5	147.7 ± 50.3	129.6 ± 49.3	121.3 ± 49.5b	0.009
ARDS severity, n (%)					0.012
Mild	19 (9.0)	8 (14.5)	6 (7.8)	5 (6.4)	0.243
Moderate	126 (60.0)	38 (69.1)	45 (58.4)	43(55.1)	0.254
Severe	65 (31.0)	9 (16.4)	26 (33.8)^a^	30 (38.5)^b^	0.020
APACHE II score	18.9 ± 5.5	17.8 ± 5.0	18.1 ± 4.9	20.4 ± 6.0^b,c^	0.009
Tracheotomy, n (%)	49 (23.3)	9 (16.4)	24 (31.2)	16 (20.5)	0.106
Duration of invasive ventilation (h)	173.5± (72.0-301.75)	181.3± (60–229)	159± (72–228)	234± (94–372) ^b^	0.030
life support treatment, n (%)					
IABP	50 (23.8)	12 (21.8)	21 (27.3)	17 (21.8)	0.669
CRRT	69 (32.9)	20 (36.4)	29 (37.7)	20 (25.6)	0.228
ECMO	40 (19.0)	8 (14.5)	13 16.9()	19 (24.4)	0.304
Length of stay, median (IQR)					
ICU LOS	9.0 (4.0–15.0)	6.0 (2.0–13.0)	10.0 (4.5–14.5)	10.5 (6.0-17.3) ^b^	0.011
Hospital LOS	25.0 (16.0-39.3)	27.0 (21.0–42.0)	24.0 (17.0–37.0)	21.5 (14.0–39.0)	0.096
Mortality, n (%)					
ICU mortality, n (%)	44 (21.0)	3 (5.5)	15 (19.5)	26 (33.3) ^b^	< 0.001
Hospital mortality, n (%)	88 (41.9)	16 (29.1)	31 (40.3)	41 (52.6) ^b^	0.024

Short CPB time = 0 < CPB < 173 min, long CPB time = CPB 173 ≥ minutes

WBC = white blood cell, Hb = hemoglobin, BUN = blood urea nitrogen, IABP = intra-aortic balloon pump, CRRT = continuous renal replacement therapy, ECMO = extracorporeal membrane oxygenation, ICU = intensive care unit

had higher left ventricular ejection fraction (LVEF), higher proportion in hypertension, lower body mass index (BMI), lower proportion in diabetes mellitus and medicine use, including ACEIs, beta-blockers, aspirin, and statins. For surgery type, we found that patients who underwent emergency surgery inclined to experience long CPB time. As for postoperative part, patients with long CPB time had lower level of hemoglobin and oxygen index, longer time of incubation and ICU stay, more severe in ARDS, and higher level of sodium, potassium, blood urea nitrogen (BUN), lactate, central venous pressure (CVP), APACHE II score after cardiac surgery.

### Association between CPB time and clinical outcomes

Before adjusting for covariates, we firstly evaluated the impact of CPB time and other risk factors on ICU and in-hospital mortality via univariate logistic regression models (Tables 3 and 4). The results demonstrated that the patients with long CPB time had significantly higher mortality risk in both ICU mortality (OR 8.67, 95%CI 2.47–30.41, *P* = 0.001) and in-hospital mortality (OR 2.70, 95%CI 1.30–5.62, *P* = 0.008) compared with non-CPB patients.

**Table 3:** Univariable logistic regression analysis of general characteristics for ICU and hospital mortality

	ICU mortality			Hospital mortality	
	Odds ratio (95% CI)	*P* value		Odds ratio (95% CI)	*P* value
CPB time (ref, non CPB (CPB = 0))					
Short CPB time	4.19 (1.15–15.28)	0.030		1.64 (0.79–3.44)	0.188
Long CPB time	8.67 (2.47–30.41)	0.001		2.70 (1.30–5.62)	0.008
Male (ref, female)	0.53 (0.25–1.16)	0.111		0.70 (0.39–1.27)	0.245
Age category (ref, ≤ 45 years)					
45 to 70 years	0.64 (0.28–1.44)	0.280		0.71 (0.34–1.47)	0.356
≥ 70 years	0.33 (0.10–1.06)	0.062		0.75 (0.31–1.83)	0.524
BMI category (ref, Normal (18.4 to 23.9))					
Underweight (≤ 18.4)	2.00 (0.53–7.54)	0.306		2.05 (0.57–7.41)	0.271
Overweight (23.9–27.9)	1.10 (0.51–2.35)	0.825		0.60 (0.32–1.13)	0.115
Obesity (≥ 27.9)	0.32 (0.32–2.37)	0.792		0.46 (0.20–1.03)	0.060
Comorbidity					
Smoking	0.91 (0.46–1.81)	0.790		1.13 (0.64–1.99)	0.671
Hypertension	1.44 (0.73–2.44)	0.291		1.02 (0.59–1.77)	0.949
Diabetes	0.96 (0.42–2.19)	0.927		0.84 (0.43–1.66)	0.621
Cerebrovascular disease	0.62 (0.20–1.90)	0.405		0.95 (0.42–2.15)	0.896
Chronic lung disease	2.24 (0.71–7.05)	0.169		1.04 (0.35–3.12)	0.940
Coronary heart disease	1.10 (0.56–2.15)	0.791		1.05 (0.60–1.82)	0.878
Valve disease	1.90 (0.41–2.89)	0.862		1.13 (0.50–2.54)	0.775
Preoperative LVEF (ref, LVEF ≥ 50%)	2.05 (0.77–5.45)	0.148		1.60 (0.65–3.95)	0.308
NYHA	1.49 (0.76–2.94)	0.247		2.46 (1.38–4.39)	0.002
Preoperative drug therapy					
ACEI	0.75 (0.35–1.60)	0.460		1.00 (0.55–1.83)	0.995
β-blocker	0.97 (0.50–1.89)	0.930		0.76 (0.44–1.32)	0.325
CCB	1.41 (0.70–2.83)	0.341		1.02 (0.56–1.84)	0.956
Aspirin	0.99 (0.35–2.83)	0.988		1.20 (0.51–2.82)	0.679
Statins	0.39 (0.17–0.90)	0.027		0.67 (0.37–1.21)	0.180
Operation type (ref, CABG)					
Valve replacement	1.96 (0.79–4.84)	0.145		2.04 (1.00-4.16)	0.049
Ascending aorta replacement	1.71 (0.70–4.21)	0.240		1.05 (0.52–2.14)	0.885
Other	2.57 (0.56–11.82)	0.225		2.80 (0.71–10.97)	0.141
Operation time	1.17 (1.05–1.32)	0.007		1.08 (0.98–1.20)	0.139
Aortic clamping time	1.00 (0.99–1.01)	0.117		1.00 (0.99-1.00)	0.238
Blood loss (ref, 800 ml)	1.21 (0.62–2.35)	0.581		1.08 (0.98–1.20)	0.139
RBC infusion (U)	1.01 (0.98–1.04)	0.419		1.10 (0.64–1.91)	0.725

Short CPB time = 0 < CPB < 173 min, long CPB time = CPB 173 ≥ minutes

**Table 4:** Univariable logistic regression analysis of postoperative characteristics for ICU and hospital mortality

	ICU mortality			Hospital mortality	
	Odds ratio (95% CI)	*P* value		Odds ratio (95% CI)	*P* value
WBC (ref, 4–10)					
WBC < 4	2.25 (0.16–31.33)	0.546		1.33 (0.10-17.55)	0.827
WBC > 10	0.87 (0.27–2.86)	0.823		1.83 (0.67–4.99)	0.240
Hemoglobin (ref, ≥ 110 g/L)					
Hb < 110 g/L	3.90 (0.51–30.98)	0.190		1.09 (0.37–3.18)	0.877
Sodium (mmol/L)					
< 135	0.90 (0.24–3.44)	0.878		1.07 (0.38–2.99)	0.903
> 145	1.48 (0.74–2.96)	0.270		1.51 (0.85–2.68)	0.164
Potassium (mmol/L)					
< 3.5	0.36 (0.10–1.24)	0.106		0.50 (0.22–1.14)	0.099
> 5.5	2.28 (0.87–5.95)	0.094		2.77 (1.06–7.25)	0.038
BUN (mg/dl)					
< 9	5.00 (0.28–88.53)	0.272		2.00 (0.12–34.10)	0.632
> 20	1.40 (0.60–3.26)	0.436		1.60 (0.81–3.14)	0.176
Lactate (mmol/L)	1.07 (0.99–1.15)	0.077		1.04 (0.98–1.10)	0.243
Central venous pressure (cmH_2_O)					
> 12	1.69 (0.84–3.40)	0.139		2.82 (1.53–5.18)	0.001
Postoperative EF (ref, ≥ 50%)	2.46 (1.24–4.86)	0.010		2.80 (1.54–5.10)	0.001
ARDS severity					
Moderate	5.84 (0.75–45.35)	0.92		1.12 (0.45–2.79)	0.802
Severe	8.13 (1.02–65.01)	0.041		1.17 (0.44–3.09)	0.757
APACHE II	1.09 (1.02–1.16)	0.009		1.06 (1.00-1.11)	0.045
Life support treatment					
IABP	0.93 (0.42–2.04)	0.850		0.81 (0.42–1.55)	0.522
CRRT	1.07 (0.53–2.17)	0.845		1.20 (0.67–2.15)	0.535
ECMO	1.12 (0.49–2.57)	0.789		1.32 (0.66–2.65)	0.426

We also found that lower postoperative LVEF, and higher APACHE II score were associated with increased ICU and in-hospital mortality risk (*P* < 0.05). Longer time of operation was a risk factor for ICU mortality. Three or higher grade of NYHA, increased level of potassium and CVP, and valve replacement were related to higher hospital mortality (*P* < 0.05). Interestingly, use of statins was a protective factor for ICU mortality in our results.

**Fig. 2:**
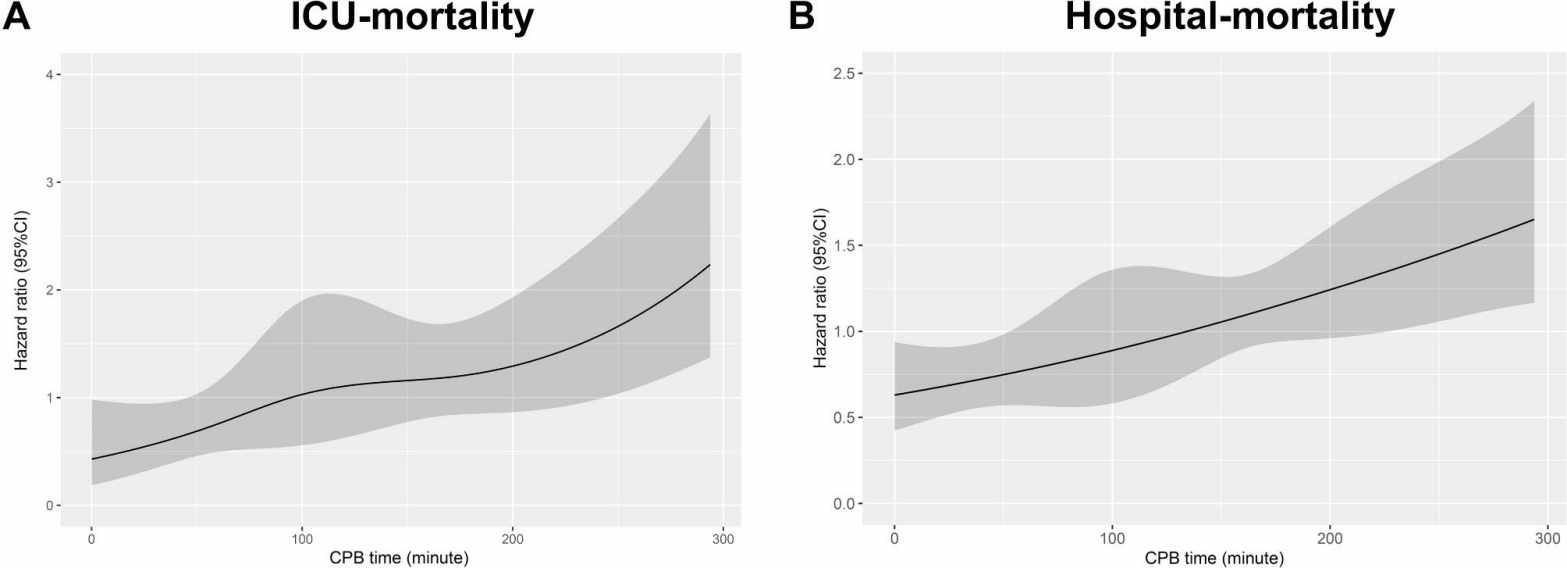
Construction of smooth curve for the relationship between CPB time and ICU (**A**) and hospital (**B**) mortality for ARDS patients by restricted cubic spline

**Fig. 3:**
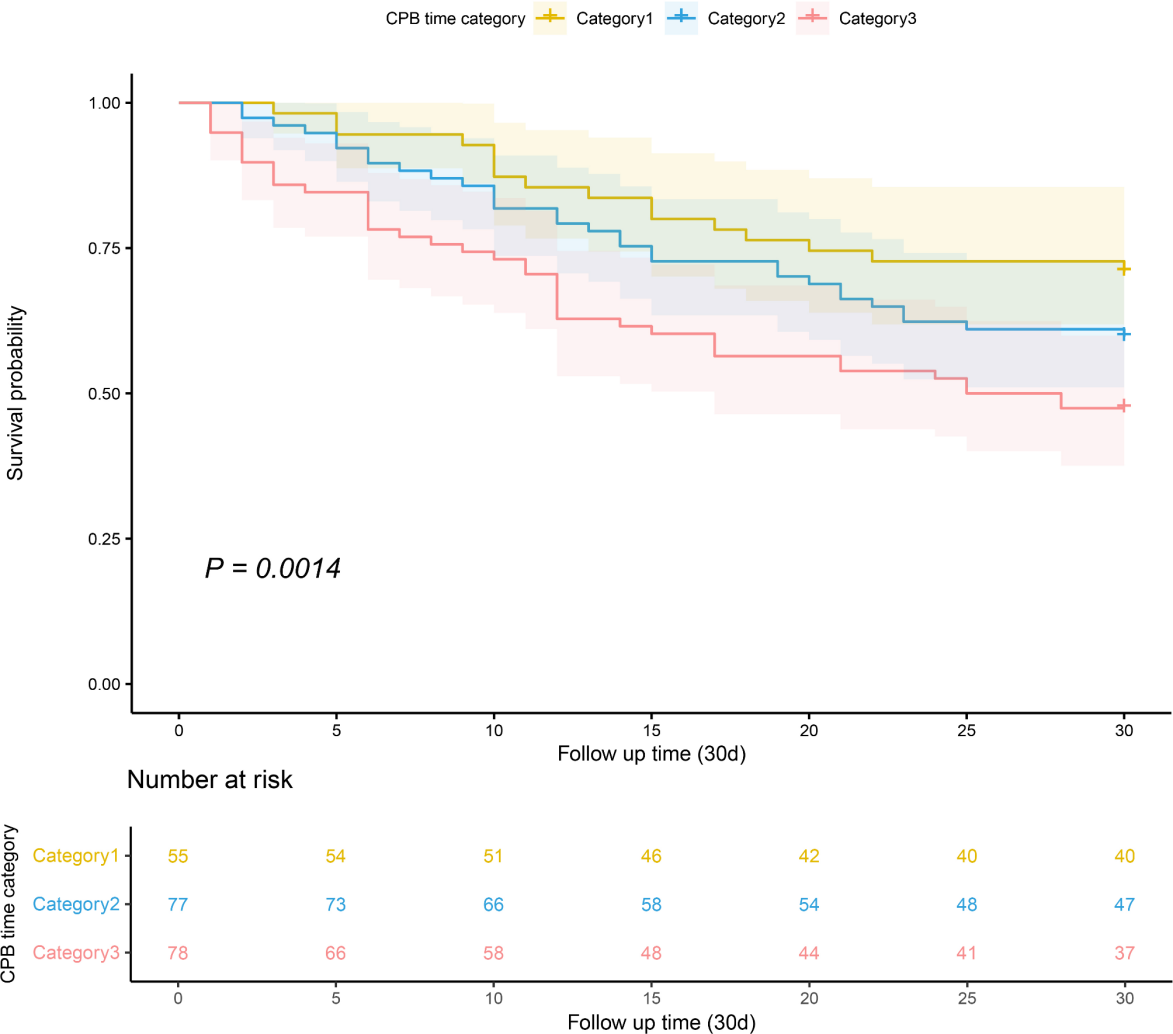
Kaplan-Meier survival curves for postoperative patients with ARDS according to CPB time. x-Axis: survival time (d). y-Axis: cumulative survival probability. ARDS, acute respiratory distress syndrome; CPB, cardiopulmonary bypass

Considering CPB time as a continuous variable, we investigated the crude relationship between CPB time and short-term mortality by utilizing RCS model, as shown in Fig. 2. It clearly indicated that longer time of CPB was associated with higher risk of ICU mortality and in-hospital mortality. K-M survival curves at 30-day demonstrated (Fig. 3) that patients with longer time of CPB presented lower survival rate and shorter survive time (log-rank test, *P* = 0.014).

Furthermore, multivariable Cox regression analysis indicated a close relationship between CPB group and short-term survival (Table 5). In the crude model, patients with long CPB time had higher risks of ICU (HR 7.17, 95%CI 2.17–23.69, *P* = 0.001) and in-hospital mortality (HR 2.23, 95% CI 1.25–3.98, *P* = 0.006) compared with non-CPB patients. After adjusting for all clinically relevant factors, patients with short or long CPB time remained markedly increased mortality in ICU (model2: short CPB time, HR 15.12, 95%CI 3.07–74.48, *P* = 0.001; long CPB time, HR 35.29, 95% CI 6.66-181.71, *P* < 0.001) and hospital (model 2: short CPB time, HR 3.21, 95%CI 1.11–9.32, *P* = 0.032; long CPB time, HR 6.51, 95% CI 1.97–21.47, *P* = 0.002). we also considered CPB time as a continuous variable for multivariable Cox regression analysis, the results showed (Table 6) the risk of ICU mortality increased by 13.3% (95%CI 7.3-19.7%, *P* < 0.001) and in-hospital mortality increased by 9.3% (95%CI 4.7-14.1%, *P* < 0.001) for every 10 min increment in CPB time after adjusting for potential factors.

**Table 5:** Multivariable Cox regression analysis for ICU and hospital mortality grouped by CPB category

Variable	Crude			Model 1			Model 2	
	HR (95%CIs)	*P* value		HR (95%CIs)	*P* value		HR (95%CIs)	*P* value
**ICU mortality**								
Non-CPB	1 (ref)			1 (ref)			1 (ref)	
Short CPB time	3.74 (1.08–12.91)	0.037		3.38 (0.96–11.91)	0.058		15.12 (3.07–74.48)	0.001
Long CPB time	7.17 (2.17–23.69)	0.001		6.49 (1.91–20.09)	0.003		35.29 (6.66-181.71)	< 0.001
**Hospital mortality**								
Non-CPB	1 (ref)			1 (ref)			1 (ref)	
Short CPB time	1.48 (0.81–2.71)	0.203		1.27 (0.68–2.38)	0.449		3.21 (1.11–9.32)	0.032
Long CPB time	2.23 (1.25–3.98)	0.006		2.14 (1.17–3.92)	0.014		6.51 (1.97–21.47)	0.002

Short CPB time = 0 < CPB < 173 min, long CPB time = CPB 173 ≥ minutes

Model1: The results were adjusted by age, gender, BMI;

Model2: The results were adjusted by age, gender, BMI, smoking, hypertension, diabetes, cerebrovascular disease, chronic lung disease, coronary heart disease, valve disease, ACEI, β_blocker, CCB, aspirin, statins, preoperative LVEF, NYHA, operation type, aortic clamping time, operation time, blood loss, transfusion of RBCModel1: The results were adjusted by age, gender, BMI;

**Table 6:** Multivariable Cox regression analysis for ICU and hospital mortality grouped by CPB time

Variable	Crude			Model 1			Model 2	
	HR (95%CIs)	*P* value		HR (95%CIs)	*P* value		HR (95%CIs)	*P* value
**ICU mortality**								
CPB time/ per10 minutes	1.06 (1.03–1.09)	< 0.001		1.06 (1.03–1.09)	< 0.001		1.13 (1.07–1.20)	< 0.001
**Hospital mortality**								
CPB time/ per 10 min	1.04 (1.02–1.06)	< 0.001		1.041 (1.2–1.07)	< 0.001		1.093 (1.05–1.11)	< 0.001

Model1: The results were adjusted by age, gender, BMI;

Model2: The results were adjusted by age, gender, BMI, smoking, hypertension, diabetes, cerebrovascular disease, chronic lung disease, coronary heart disease, valve disease, ACEI, β_blocker, CCB, aspirin, statins, preoperative LVEF, NYHA, operation type, aortic clamping time, operation time, blood loss, transfusion of RBC

### ROC curve analysis

**Fig. 4:**
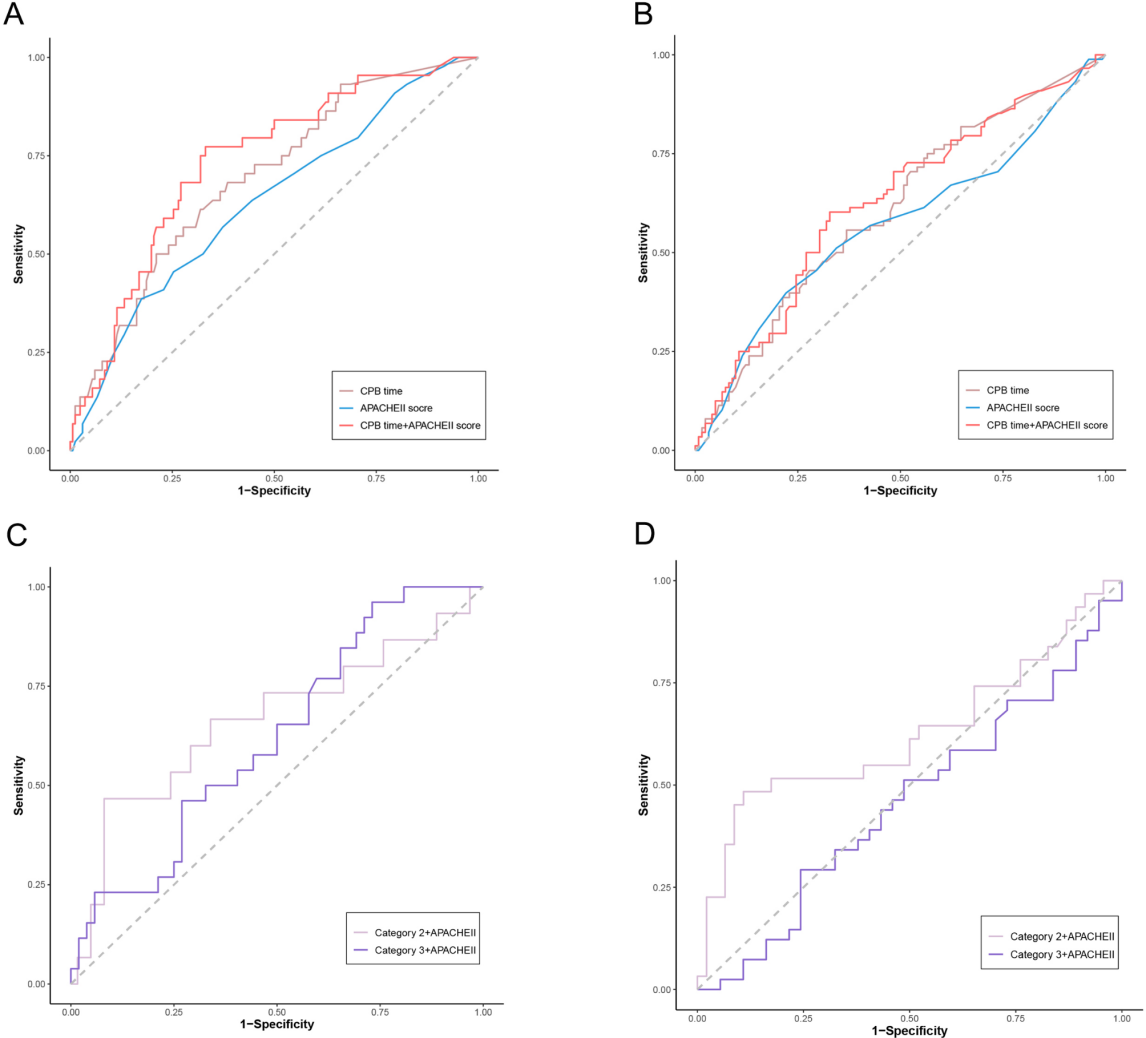
ROC curves for predicting ICU and hospital mortality in ARDS patients with CPB. (**A**, **B**) The ability of CPB time and APACHE II score to predict ICU and hospital mortality. (**C**, **D**) The ability of combined CPB time and APACHEII score in predicting ICU and hospital mortality

APACHE II score is widely used in helping clinicians evaluate the disease severity and predict mortality. We performed ROC analysis to test and compare the power of CPB time and APACHE II score for predicting short-term mortality. For the ICU mortality, the area under the ROC curve (AUC) was 0.693 (95% CI: 0.608–0.778, *P* < 0.001) for CPB time, 0.631 (95% CI: 0.538–0.724, *P* = 0.008) for APACHE II score (Fig. 4A). and the AUC could reach 0.733 (95% CI: 0.652–0.815, *P* = 0.003) for predicting ICU mortality after combing the CPB time and APACHEII score. For hospital mortality, the AUC was 0.610 (95% CI: 0.533–0.687, *P* < 0.001) for CPB time, 0.570 (95% CI: 0.489–0.651, *P =* 0.085) for APACHE II score, and 0.626 (95% CI: 0.548–0.703, *P* = 0.005) for combined indictors. (Fig. 4B). Furthermore, we tested the predictive value of short CPB time group and long CPB time group for the mortality after combing with APACHEII score. The results (Fig. 4C **and D**) showed the AUC of combined short CPB time group and APACHEII score was 0.663 (95% CI: 0.486–0.841, *P* = 0.024) for predicting ICU mortality and 0.623 (95% CI: 0.486–0.763, *P* = 0.039) for predicting in-hospital mortality, the AUC of combined long CPB time group and APACHEII score was 0.621 (95% CI: 0.493–0.749, *P* = 0.041) for predicting ICU mortality and 0.458 (95% CI: 0.328–0.587, *P* = 0.068) for predicting in-hospital mortality. The optimal cut-off value of CPB time were 160.5 min for ICU mortality with a sensitivity of 68.2% and a specificity of 61.5% and for hospital mortality with a sensitivity of 55.7% and a specificity of 63.1%.

## Discussion

This study was to reveal the quantitative association between CPB time and clinical outcomes in ARDS patients after cardiac surgery. Firstly, patients with longer time of CPB were inclined to have worse postoperative manifestations (such as lower postoperative LVEF and oxygen index, higher score of APCHE II, and longer duration of mechanical ventilation) and poorer outcomes including higher ICU mortality and in-hospital. Secondly, for each ten minutes increment in CPB time, the hazards of a worse outcome increased by 13.3% for ICU mortality and 9.3% for in-hospital mortality after adjusting for potential factors. Finally, CPB time presented more satisfactory power to predict ICU mortality and in-hospital mortality for postoperative patients with ARDS compared with APCHEII score, and the optimal cut-off value of CPB time was 160.5 min.

Postoperative ARDS after cardiac surgery with CPB is a rare but fatal complication that prolongs the duration of mechanical ventilation and hospital stay and even leads to death [12]. CPB is a widely known risk factor for the occurrence of postoperative ARDS [8]. It is generally recognized that exposure of blood to abnormal surfaces, as in the case of CPB, can trigger exaggerated inflammatory response and induce the lung dysfunction even occurring ARDS in patients undergoing cardiac surgery [13]. Many previous studies paid attention to the mechanism of ARDS induced by CPB including activation of complement, abnormal immune reaction to protamine, and dysfunction of platelets [7, 14]. However, there are few research focused on the specific association between CPB time and the postoperative outcomes in patients who undergo cardiac surgery.

In our study, the total ICU mortality and in-hospital mortality was respectively 21.0% and 41.9% in all ARDS patients after cardiac surgery, which was consistent with previous studies that the mortality was approximately 15–50% [15]. However, the mortality rate increased significantly as CPB time prolonged, that is the ICU mortality in patients with long CPB time (CPB time ≥ 173 min) was 33.3% and in-hospital mortality could reach 52.6%. Furthermore, the longer CPB time extended the length of ICU stay from 6 to 10.5 days. Moreover, we found that patients with non or short time of CPB had higher incidence of diabetes mellitus, lower incidence of hypertension, more use of cardiac-related drugs, and better cardiac function. We speculated it because most of patients with non or short time of CPB were diagnosed with coronary heart diseases (CHDs) which is closely associated with the incidence of diabetes mellitus, and patients undergoing long time CPB often suffer from aortic problems. It has been confirmed that the incidence of aortic disease is related to hypertension [16], and these patients tend to have better cardiac function than those with CHDs or valve disease [17]. In postoperative part, lower level of hemoglobin and oxygen index, higher severity of ARDS and higher level of sodium, potassium, BUN, lactate, CVP, APACHE II score, and longer time of incubation were the corresponding reflection of systemic inflammation and poor prognosis. Bendjelid et al. [18] proposed that the lung was a source of lactate production during CPB because lung parenchyma was hypo-perfused in extracorporeal circulation, and the concentration of the lactate was associated with the degree of hypoxemia. Yang [19] found that high level of CVP was related to acute kidney injury after cardiovascular surgery and it was also an independent risk factor for all-cause mortality.

We confirmed that CPB was an independent risk factor in both ICU mortality and in-hospital mortality in ARDS patients after cardiac surgery, and for each ten minutes increment in CPB time, the ICU mortality increased by 13.3% for and in-hospital mortality increased by 9.3% for after adjusting for potential factors. In addition to CPB time, we also found that lower postoperative LVEF, and higher APACHE II score were associated with increased short-term mortality risk (*P* < 0.05). Correspondingly, Christenson et al. [20] and Kaul et al. [21] have also reported that low postoperative cardiac output such as LVEF < 40%, preoperative NYHA class 3 and 4 were independent predictors for adult ARDS after cardiac surgery. Valve surgery was another independent risk factor in hospital mortality in our data, previous study has reported that the incidence of ARDS approached 8.1% after valve surgery, and tricuspid valve replacement was regarded as an independent risk factor for ARDS [22]. Remarkably, it showed that utilization of statins was a protective factor on ICU mortality in this study. Statins have been considered as a treatment for ARDS because their potential anti-inflammatory function. Jacobson et al. [23] proposed that statins could reduce the inflammation in both pulmonary and extrapulmonary organs and prevent exacerbation of lung injury in murine models. A phase 2 randomized controlled trial (RCT) showed treatment with simvastatin was safe and associated with an improvement of organ dysfunction in ARDS patients [24]. But in the Statins for Acutely Injured Lungs from Sepsis (SAILS) trial, there was no significant difference between rosuvastatin and placebo in 60-day mortality and ventilation-free days to day28 [25]. Our results indicated that utilization of statins was a protective factor for ICU mortality for postoperative ARDS, we thought it might be related to cardiac disease itself, we will increase the number of cases to verify this result.

APACHE II score is a widely used tool that can help evaluated disease severity and predict prognosis. Huber et al. [26] showed that AUC for the APACHEII score was 0.667 and for the SOFA score was 0.763 in predicting the 28-days-mortality for ARDS patients. Basile-Filho’s [27] study proposed the AUC was 0.850 for the APACHEII score regarding hospital mortality in surgical critically ill patients. We used APACHE II score as a reference to evaluate the ability of CPB time in predicting the mortality of patients with ARDS after cardiac surgery. The results indicated that CPB time had a higher efficiency to predict mortality compared with APACHEII score, and the optimal cut-of value for ICU and hospital mortality were 160.5 min. Chen [28] has reported that CPB time ≥ 132 min was associated with delayed recovery in patients after cardiac surgery. Madhavan [29] suggested that CPB time > 180 min was not only associated with postoperative outcomes including prolonged ICU stay, hospital stay, mechanical ventilation and reoperation but also could predict 1-year mortality. Additionally, our data suggested CPB time had a high positive predictive value in ICU mortality, which mean we should increase vigilance against the occurrence of ARDS for the patients underwent long time of CPB. However, the negative predictive value was relatively low. It may be that the onset of ARDS is a complex process that induced by multiple factors, not only CPB.

According to our best knowledge, this is the first report that reveal the quantitative relationship between CPB time and clinical outcomes for patients with ARDS after cardiac surgery. Although previous studies had confirmed that CPB was a risk factor for developing ARDS after cardiac surgery, but few studies provide a quantitative relationship between CPB time and postoperative outcomes of those ARDS patients. Our findings exhibited that the longer time of CPB was associated with more detrimental clinical presentations and higher mortality risk in ARDS patients after cardiac surgery, and for each ten minutes increment in CPB time, the ICU mortality increased by 13.3% for and in-hospital mortality increased by 9.3%. We also provided an optimal cut-off time, that is 160.5 min for predicting ICU mortality.

There were several limitations in our research. First, there was no clear standard about how long can be defined as long CPB time, our definition is according to the median time of all patients which may limit the comparability of the results. Second, some proinflammatory targets such as IL-6, PCT or CRP were not contained in our study, so we failed to clearly explicit the relationship between CPB and systemic inflammatory response. Third, the number of patients among three categories was imbalanced, which may influence the reliability of results. Finally, the sensitivity and specificity values of CPB time to predict the mortality of postoperative ARDS were not sufficiently high, so predictions could not rely solely on CPB time.

## Conclusion

Long time of CPB was not only associated with detrimental postoperative manifestations, but also acted as an independent risk factor for increased ICU mortality and in-hospital mortality in ARDS patients underwent cardiac surgery. CPB time had a satisfactory power to predict short-term mortality compared with APACHEII score. However, more prospective studies are still needed to prove the reliability of CPB time as an indicator for predicting prognosis in the patients with ARDS after cardiac surgery.

## Data Availability

The data in this study are available from the corresponding author on reasonable request.
